# Retrospective Cohort Study on the Limitations of Direct-to-Consumer Genetic Screening in Hereditary Breast and Ovarian Cancer

**DOI:** 10.1200/PO.22.00695

**Published:** 2023-08-03

**Authors:** Neelam V. Desai, Elizabeth D. Barrows, Sarah M. Nielsen, Kathryn E. Hatchell, Michael J. Anderson, Eden V. Haverfield, Blanca Herrera, Edward D. Esplin, Anneke Lucassen, Nadine M. Tung, Claudine Isaacs

**Affiliations:** ^1^ Levine Cancer Institute, Atrium Health, Charlotte, NC; ^2^ Division of Hematology-Oncology, MedStar Georgetown University Hospital, Washington, DC; ^3^ Georgetown University Medical Center, Lombardi Comprehensive Cancer Center, Washington, DC; ^4^ Invitae Corp, San Francisco, CA; ^5^ Department of Clinical Ethics and Law at Southampton, University of Southampton, Southampton, United Kingdom; ^6^ Welcome Centre for Human Genetics, University of Oxford, Oxford, United Kingdom; ^7^ Division of Hematology-Oncology, Department of Medicine, Beth Israel Deaconess Medical Center, Boston, MA; ^8^ Harvard Medical School, Boston, MA

## Abstract

**PURPOSE:**

Among cancer predisposition genes, most direct-to-consumer (DTC) genetic tests evaluate three Ashkenazi Jewish (AJ) founder mutations in *BRCA1/2*, which represent a small proportion of pathogenic or likely pathogenic variants (PLPV) in cancer predisposing genes. In this study, we investigate PLPV in *BRCA1/2* and other cancer predisposition genes that are missed by testing only AJ founder *BRCA1/2* mutations.

**METHODS:**

Individuals were referred to genetic testing for personal diagnoses of breast and/or ovarian cancer (clinical cohort) or were self-referred (nonindication-based cohort). There were 348,692 participants in the clinical cohort and 7,636 participants in the nonindication-based cohort. Both cohorts were analyzed for *BRCA1/2* AJ founder mutations. Full sequence analysis was done for PLPV in *BRCA1/2*, *CDH1*, *PALB2*, *PTEN*, *STK11*, *TP53*, *ATM*, *BARD1*, *BRIP1*, *CHEK2* (truncating variants), *EPCAM*, *MLH1*, *MSH2/6*, *NF1*, *PMS2*, *RAD51C/D*, and 22 other genes.

**RESULTS:**

*BRCA1/2* AJ founder mutations accounted for 10.8% and 29.7% of *BRCA1/2* PLPV in the clinical and nonindication-based cohorts, respectively. AJ founder mutations accounted for 89.9% of *BRCA1/2* PLPV in those of full AJ descent, but only 69.6% of those of partial AJ descent. In total, 0.5% of all individuals had a *BRCA1/2* AJ founder variant, while 7.7% had PLPV in a high-risk breast/ovarian cancer gene. For non-AJ individuals, limiting evaluation to the AJ founder *BRCA1/2* mutations missed >90% of mutations in actionable cancer risk genes. Secondary analysis revealed a false-positive rate of 69% for PLPV outside of non-AJ *BRCA 1/2* founder mutations.

**CONCLUSION:**

DTC genetic testing misses >90% of *BRCA1/2* PLPV in individuals of non-AJ ancestry and about 10% of *BRCA1/2* PLPV among AJ individuals. There is a high false-positivity rate for non-AJ *BRCA 1/2* PLPV with DTC genetic testing.

## Teaser

Direct-to-consumer genetic testing has very limited utility in screening for mutations in breast cancer genes.

## BACKGROUND

Direct-to-consumer (DTC) genetic testing allows individuals to purchase access to their personal genetic information without input from a health care professional. It is marketed as a way for individuals to gain a better understanding of their genetic information. DTC genetic testing was introduced in the 2000s and has quickly gained popularity among the public. In 2019, more than 26 million individuals had taken at-home genetic tests, and that number continues to rise by the year.^1^ Part of its popularity stems from the fact that DTC testing may provide individuals with information about susceptibility for various medical conditions, including cancer risk. However, there has been growing controversy regarding the clinical utility of DTC testing in assessing patients' risk for developing cancer.

**CONTEXT**

**Key Objective**
We investigate the proportion of pathogenic or likely pathogenic variants (PLPV) that are missed by testing only the three Ashkenazi Jewish (AJ) founder *BRCA1/2* variants usually tested in direct-to-consumer (DTC) testing.
**Knowledge Generated**
We found that DTC testing misses >90% of actionable cancer risk genes in individuals of non-AJ descent. Even in AJ individuals, DTC genetic testing misses 10% of *BRCA1/2* PLPV. There is a high false-positivity rate of up to 69% for non-AJ *BRCA1/2* PLPV with DTC genetic testing.
**Relevance**
Our study highlights that DTC genetic testing has limited utility as a screening tool for identifying patients at risk for hereditary breast or ovarian cancer, especially in those of non-AJ descent. This is further underscored by the high false-positivity rate we found with DTC genetic testing. Greater education is needed to both patients and providers regarding the limitations of DTC genetic testing.

There have been few systematic analyses that have looked at the impact of DTC genetic testing on consumers' health perceptions. False-positive and false-negative DTC genetic testing results may provide an individual with either unnecessary worry or false reassurance regarding their risk for certain medical conditions.^2^ Studies have shown that consumers experience stress when they receive unexpected results, especially results that indicate an increased risk for cancer.^3^ Most medical information from DTC genetic test results is accompanied by a disclaimer that the information provided is not intended for medical use without confirmation in a clinical laboratory and recommends that consumers discuss their results with a health care provider. However, when consumers approach their health care providers with DTC genetic testing results, many of these providers lack adequate awareness to order confirmatory genetic testing or knowledge to interpret clinical-grade genetic test results, particularly those pertaining to cancer risk.^4^

Although DTC genetic testing is an accurate and cost-effective way to analyze common genetic variants within a population to determine traits such as ancestry, this testing performs poorly when genotyping rare genetic variants such as those that can occur in cancer predisposition genes.^5^ Additionally, false positives can result from analytic issues or clinical misclassification. Unlike clinical laboratory grade testing, DTC genetic testing companies are not required to be Clinical Laboratory Improvement Amendments–certified or College of American Pathologists–accredited, which provide strict standards for diagnostic testing.^6^ This further underscores differences in the rigor of testing requirements for DTC companies as opposed to commercial laboratories. One study found that of 49 samples assessed, 40% of pathogenic or likely pathogenic variants (PLPV) identified in DTC raw data were clinical false positives.^7^ This necessitates further evaluation into the clinical accuracy of DTC genetic testing.

In 2018, the US Food and Drug Administration (FDA) authorized 23andMe (San Francisco, CA) to market DTC testing limited to the three *BRCA1/2* founder mutations most common in people of Ashkenazi Jewish (AJ) descent: *BRCA1* 185delAG, *BRCA1* 5382insC, and *BRCA2* 6174delT.^8^ The presence of any of these three pathogenic *BRCA1/2* variants is considered diagnostic of hereditary breast and ovarian cancer (HBOC) syndrome. These three *BRCA1/2* founder mutations are identified in approximately 2%-2.5% of unselected AJ individuals.^9^ One study showed that in individuals of AJ descent, these three founder mutations account for 87% and 92.8% of the mutations in *BRCA1* and *BRCA2*, respectively.^10^ However, these three mutations account for the minority of PLPV in *BRCA1/2* in the non-AJ population.^11^ This leads to the concern that individuals who order these tests may have a false sense of security regarding their cancer risk upon receiving a negative DTC test result. The FDA has also reported that only a small percentage of Americans carry one of these mutations. However, on the basis of their review in 2018, 23andMe provided sufficient data to demonstrate that their DTC testing for these three founder mutations is accurate and reproducible, and that these results can be easily understood by its customers. On the basis of these findings, the FDA approved this DTC genetic test for public use.

With an increasing number of individuals ordering limited-variant DTC genetic screening, the impact of DTC genetic screening on individuals' health perceptions is an emerging concern. The purpose of this study is to assess the potential limitations of DTC genetic screening for HBOC, as well as for other hereditary cancer syndromes.

## METHODS

We investigated the PLPV missed by only analyzing the three AJ founder variants, performed by a commercial laboratory (Invitae, San Francisco, CA). The primary aim of this study was to assess the percentage of *BRCA1/2* mutations in AJ and non-AJ populations accounted for by the three AJ *BRCA1/2* founder mutations included in DTC screening. As a secondary aim, we assessed the frequency of PLPV in other cancer risk genes not tested by DTC screening limited to the three AJ *BRCA1/2* founder mutations. AJ ancestry was by self-report. An individual was considered of full AJ ancestry if they reported AJ as their sole ethnicity. They were considered of partial AJ descent if they reported AJ plus another ethnicity.

The first cohort (clinical cohort) in our study included 348,692 individuals with a reported personal history of breast and/or ovarian cancer who were referred by a health care provider for HBOC multigene panel testing. The second cohort (nonindication-based cohort) included 7,636 individuals who self-referred for genetic testing in the setting of executive health or wellness clinic offerings. They were considered ostensibly healthy on the basis of an absence of reported personal or family history of cancer. Participants with a reported family history were excluded from the nonindication-based cohort to more accurately create the clearest distinction between the two cohorts and better examine the impact of DTC genetic testing in those without any potential predispositions to HBOC. An individual’s ancestry was self-reported on the test requisition form.

The primary genetic analysis evaluated *BRCA1/2* AJ founder mutations (group 1 genes) and all PLPV in *BRCA1/2* (group 2 genes, including AJ *BRCA1/2* founder mutations) in all patients, to assess how frequently *BRCA1/2* mutations other than the AJ founder mutations would be missed in various populations.

Secondary analyses were performed on a subset of patients who underwent comprehensive genetic testing in additional cancer genes. These analyses investigated PLPV in cancer risk genes that would be missed by screening for only the three AJ founder *BRCA1/2* mutations. Secondary analyses performed included evaluating PLPV in high-risk breast cancer genes (*BRCA1/2*, *CDH1*, *PALB2*, *PTEN*, *STK11*, and *TP53*; group 3 genes), all breast or ovarian cancer risk genes (group 3 genes plus *ATM*, *BARD1*, *BRIP1*, *CHEK2* [truncating], *EPCAM*, *MLH1*, *MSH2/6*, *NF1*, *PMS2*, and *RAD51C/D*; group 4 genes), and 41 other cancer risk genes (group 5 genes) (Table 1). To avoid confounding by gene-ordering patterns, this secondary analysis was limited to those participants from the primary analysis who were tested for all 41 genes. For all individuals tested in both cohorts, full gene sequencing, as well as deletion/duplication analysis, was performed as previously described.^12^ For all analyses, the variant interpretation was performed using a refinement of the American College of Medical Genetics and Genomics criteria.^13^ Variants deemed mosaic were excluded.

**TABLE 1.**
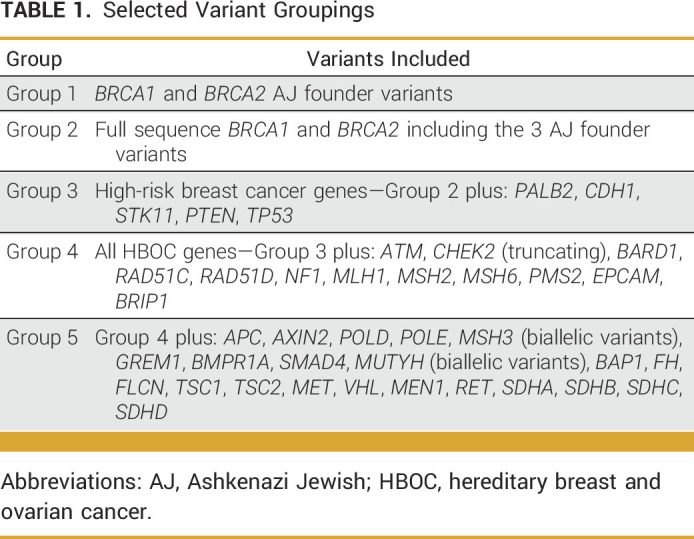
Selected Variant Groupings

A limited number of patients (n = 287) had received previous DTC screening and were subsequently referred for confirmatory testing in the clinical laboratory. Their deidentified data were analyzed and used to calculate a false-positive rate. In many (n = 217) of these cases, individuals personally subjected their DTC-obtained genetic data to DTC third-party variant interpretation platforms (eg, Promethease), which reported potential mutations in cancer predisposition genes.

## RESULTS

Our primary analysis included 356,328 individuals who underwent at least full *BRCA1/2* sequencing. Among 348,692 individuals in the clinical cohort, 1,513 (0.4%) had a *BRCA1/2* AJ founder mutation. By contrast, on full gene sequence analysis of *BRCA1/2*, 13,987 (4%) these individuals had a *BRCA1/2* PLPV. Similarly, among the 7,636 individuals in the nonindication-based cohort, only 19 (0.2%) had a *BRCA1/2* AJ founder mutation, but 64 (0.8%) had a PLPV in *BRCA1/2* on comprehensive gene sequencing. The *BRCA1/2* AJ founder mutations accounted for 10.8% of all *BRCA1/2* PLPVs in the clinical cohort, and 29.7% of *BRCA1/2* PLPVs in the nonindication-based cohort (Table 2). In the case cohort, the remaining 89.2% of *BRCA1/2* variants were accounted for by over 2,400 different variants, indicating the importance of comprehensive testing.

**TABLE 2.**
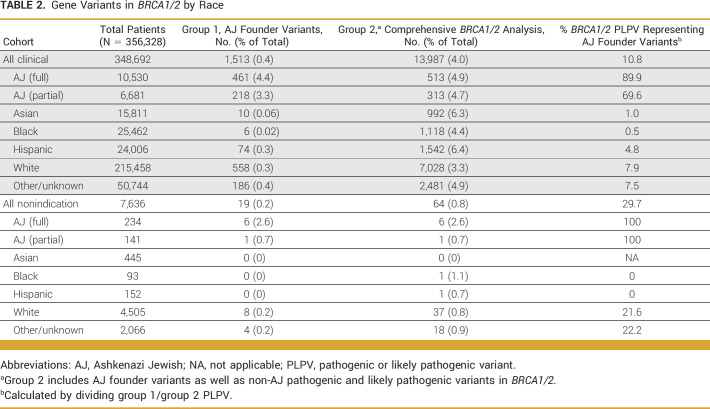
Gene Variants in *BRCA1/2* by Race

We then evaluated the likelihood of detecting an AJ founder variant in individuals stratified by self-reported ancestry. In the clinical cohort, 4.4% of individuals of full AJ ancestry and 3.3% of those with partial AJ ancestry had a *BRCA1/2* AJ founder variant. These AJ founder mutations accounted for 89.9% and 69.6% of all *BRCA1/2* PLPV among individuals with full or partial AJ descent, respectively. Among individuals of Asian, Black, Hispanic, White, or other ethnicities in the clinical cohort, the AJ founder mutations accounted for <10% of *BRCA1/2* PLPV (Table 2).

Our secondary analysis evaluated PLPV in a subset of individuals (n = 83,101) who underwent more comprehensive genetic testing beyond *BRCA1/2* (ie, those who were tested for at least the 41 cancer risk genes in the group 5 genes). Among 77,309 individuals in the clinical cohort who underwent this comprehensive multigene panel genetic testing, only 367 (0.5%) had an AJ founder *BRCA1/2* mutation. However, 6,570 (8.5%) had a PLPV in one of the 41 actionable cancer-risk genes. When stratified by gene group, 2,721 (3.5%) individuals had *BRCA1/2* PLPV on comprehensive sequencing of *BRCA1/2*; 3,489 (4.5%) individuals had a PLPV in one of the seven high-risk breast cancer genes; and 5,944 (7.7%) had a PLPV in one of the 19 breast or ovarian cancer genes (Table 3).

**TABLE 3.**
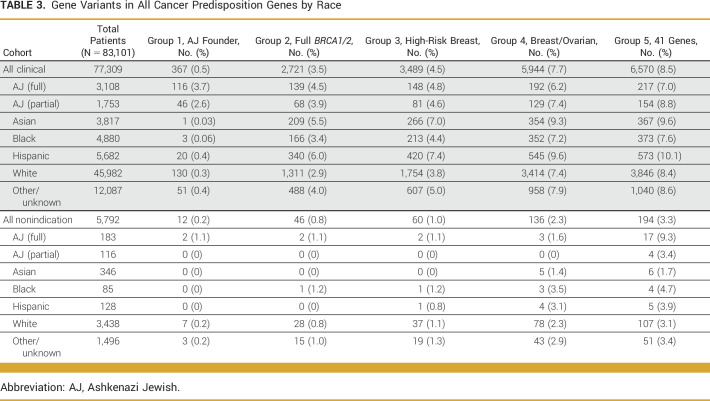
Gene Variants in All Cancer Predisposition Genes by Race

Similarly, among the 5,792 individuals within the nonindication-based cohort who had comprehensive multigene panel genetic testing, 12 (0.2%) had an AJ *BRCA1/2* founder mutation, whereas 195 (3.4%) had a PLPV in any actionable cancer risk gene. Stratified by gene group, 46 (0.8%) had a *BRCA1/2* PLPV identified through comprehensive *BRCA1/2* analysis; 60 (1.0%) had a PLPV in any of seven high-risk breast cancer genes; and 136 (2.3%) had a PLPV in any of 19 genes associated with breast or ovarian cancer (Table 3).

Raw DTC screening data were available for 287 individuals from the clinical cohort who sought confirmatory testing through a clinical laboratory after having received a reported positive result in a cancer risk gene. Among the 154 individuals who tested positive for one of the three AJ *BRCA1/2* founder mutations, the false-positive rate of DTC testing was low (n = 1; 0.6%). However, the false-positive rate of DTC testing was between 69.0% and 89.7% for reported PLPV in other cancer predisposition genes (Table 4). Among 78 patients who tested positive for a non-AJ founder *BRCA1/2* mutation, 70 were false positives (89.7%). Of note, this analysis took into account patients with *BRCA1/2* variants that were categorized as variants of unknown significance by Invitae. Additionally, although 55 patients tested positive for mutations in other cancer predisposition genes, 38% of these results were classified as false positives (n = 109).

**TABLE 4.**
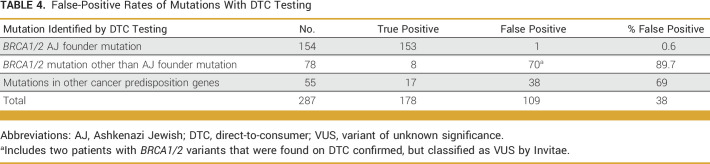
False-Positive Rates of Mutations With DTC Testing

## DISCUSSION

DTC genetic screening has become increasingly popular. However, concerns remain regarding what consumers may glean about the information these tests convey regarding their inherited risk of cancer.^7,14^ In March 2018, 23andMe received FDA authorization to market DTC screening limited to the three AJ *BRCA1/2* founder mutations. In this study, we demonstrate that evaluation of only the three AJ *BRCA1/2* founder mutations misses >90% of *BRCA1/2* PLPV in individuals of non-AJ ancestry, about 30% of those in individuals with only partial AJ ancestry, and 10% in those of full AJ ancestry. We also demonstrate the potential for a high false-positive rate for PLPV other than the AJ founder *BRCA1/2* mutations through DTC testing. The rate of nonfounder *BRCA1/2* mutations in AJ patients tested for HBOC is similar to that reported by Walsh et al.^15^ Walsh et al reported that 0.8% of full AJ patients with a diagnosis of breast cancer carried a nonfounder *BRCA1/2* variant. In our study, 0.5% (52/10,530) of patients with full AJ ancestry carried a nonfounder *BRCA1/2* mutation.

In addition, we demonstrate that only 0.5% of all individuals referred for clinical genetic testing were found to have a *BRCA1/2* AJ founder variant, while 8.5% had a PLPV in one of 41 actionable cancer risk genes. For individuals of non-AJ ancestry, limiting evaluation to only the three AJ founder *BRCA1/2* mutations missed the majority of all mutations in actionable cancer risk genes. This information has the potential to falsely reassure individuals, especially those of non-AJ descent, that they have no genetic risk of HBOC.

The number of patients with limited-variant DTC testing who underwent confirmatory clinical genetic testing in this cohort is relatively small. However, the false-positive rates for PLPV other than AJ founder *BRCA1/2* mutations are substantial and ranged from 69% to 87% (Table 4). This raises the concern that although DTC testing is accurate in evaluating the three AJ founder mutations, it is not accurate in assessing non-AJ founder mutations and mutations in other cancer predisposition genes. Patients who receive these results could be subjected to unnecessary anxiety, screening procedures, and even prophylactic surgeries by relying on these data without independent clinical confirmatory testing.

The strength of our study includes the large sample size of individuals who underwent comprehensive genetic testing. To our knowledge, this is the largest study that has evaluated how often PLPV in cancer predisposition genes would be missed by DTC testing that only evaluates the three AJ *BRCA1/2* founder mutations. A limitation of the study, however, is that the majority of individuals were referred for genetic testing because of a clinical indication such as a personal history of breast and/or ovarian cancer, thereby enriching the population for PLPV in cancer risk genes. As such, our study sample may not accurately represent the frequency of PLPV in unselected individuals who may comprise the majority of those undergoing DTC testing. It is likely that the nonindication-based cohort included in our study more closely approximates the population of individuals ordering DTC genetic testing. Additionally, ancestry was by self-report, although that is the common way these data are captured. Future studies, with comprehensive collection of family history data, could compare individuals with a pertinent family history and without a personal history to see if mutation frequencies vary with those who have a family history of HBOC. In the nonindication-based cohort, it would also have been valuable to have further information on patient motivations for pursuing genetic testing and to evaluate if test result varied on the basis of these factors.

Of note, as of 2022, after the analyses of this study were already performed, 23andMe also started providing limited-variant testing for *MUTYH* mutations Y179C and G396D, as well as the *HOXB13* G84E mutation. These genes portend an increased risk for colorectal cancer and prostate cancer, respectively.^15-17^ Our analyses include *MUTYH* as a part of our group 5 genes. However, analysis of the clinical false-negative rate for these variants in *MUTYH* and *HOXB13* was beyond the scope of the current study. This will also apply to any new genes or variants that become FDA approved for DTC testing after the publication of this study.

These data underscore that although testing for the three *BRCA1/2* AJ founder mutations has 90% sensitivity for clinically important breast cancer risk variants in those of full AJ descent, it has limited utility as a screening tool for identifying HBOC syndrome, especially among those of non-AJ or partial AJ descent. We also demonstrate that there may be a high false-positive rate for these PLPV within DTC testing, which again points to its limitations. Our data support the FDA recommendation that individuals screened with a limited DTC panel of mutations should receive confirmatory clinical genetic testing, regardless of a positive or negative result.^7^ DTC companies could consider reflexing to confirmatory testing for individuals with positive results, but at the very least should continue to provide clear guidance that their results should be confirmed and interpreted with the help of a qualified health care professional.

Additionally, greater public education is needed to increase awareness about the limitations of DTC genetic testing for HBOC screening, even among individuals of AJ ancestry. These results suggest that all hereditary cancer genetic testing and screening should include the support of a qualified health care provider who can make clinical management recommendations for patients and their family members. We also support further education for health care providers and insurance companies regarding the need for clinical confirmation after DTC genetic testing. This is especially important, given the potential for significant false-positive and false-negative rates in DTC testing, as we have demonstrated in our study. Patients should be able to easily obtain confirmatory genetic testing after a positive DTC result to better understand their risk for developing malignancy. Finally, we agree with previous recommendations that the goal should be to develop models of clinical genetic testing for cancer risk that combine the convenience of DTC testing and the comprehensiveness of clinical laboratory multigene testing with the integration of an individual's health care provider in the testing process.
